# Effects of different types of physical exercise on executive function of older adults: a scoping review

**DOI:** 10.3389/fpsyg.2024.1376688

**Published:** 2024-06-28

**Authors:** Zhidong Cai, Ruibao Cai, Li Sen

**Affiliations:** ^1^Department of Physical Education, Suzhou University of Science and Technology, Suzhou, China; ^2^School of Physical Education, Chizhou University, Chizhou, China; ^3^School of Physical Education and Health, Shanghai Lixin University of Accounting and Finance, Shanghai, China

**Keywords:** older adults, physical exercise, cognition, executive function, type of exercise

## Abstract

**Objective:**

This scoping review examined the impact of physical exercise on executive function (EF) in older adults and investigated the moderating effects of exercise types.

**Methods:**

We systematically searched four electronic databases for randomized controlled trials (RCTs) investigating the effects of exercise on EF, published until November 26, 2023. The proportions of positive and null/negative effects across all studies were calculated.

**Results:**

In total, 91 studies were included in the analysis. Among these, 27 (29.7%) studies employed aerobic exercise interventions for older adults’ EF, with 19 (70.4%) studies reporting positive effects. Additionally, 18 (19.8%) studies utilized strength exercise interventions for older adults’ EF, with 15 (83.3%) studies demonstrating positive benefits. Furthermore, 32 (35.2%) studies employed coordination exercise interventions for older adults’ EF, with 25 (78.1%) studies showing positive benefits. Similarly, 30 (33%) studies applied mixed exercise interventions for older adults’ EF, with 25 (83.3%) studies indicating positive benefits.

**Conclusion:**

Overall, all four types of physical exercise enhance EF in older adults, with mixed exercises being the most effective.

## Introduction

The global population of older adults has experienced a sharp increase, currently standing at approximately 700 million, constituting roughly 9.1% of the world population. By 2050, this figure is projected to surpass two billion, comprising 21% of the global population (Nations, 2015). The rapid expansion of the aging population has highlighted the prevalence of age-related cognitive disorders, typically associated with diminished quality of life and escalating healthcare expenditures. Consequently, preserving brain health and mitigating the decline of cognitive function have emerged as pressing imperatives in aging societies (Cai et al., 2021).

Accumulating research has demonstrated that physical exercise is crucial for delaying cognitive decline in older adults. A large number of cross-sectional studies have identified a positive correlation between physical exercise and cognitive function (Engeroff et al., 2018; Gogniat et al., 2021, 2022). Intervention studies and systematic reviews have indicated that physical exercise improves the cognitive function of older adults (Biazus-Sehn et al., 2020; Chen et al., 2020; Sanders et al., 2020), especially in terms of executive function (EF) (Colcombe and Kramer, 2003).

EF is a high-level cognitive function characterized by a complex structure, encompassing inhibition, working memory, switching, planning, and problem-solving abilities (Miyake et al., 2000; Diamond, 2013). The decline of executive function indicates a greater risk of mild cognitive impairment (MCI) and dementia in later years (Marshall et al., 2011). While there is a consensus on the benefits of physical exercise in improving executive function (Northey et al., 2017; Falck et al., 2019), there remains controversy regarding the most effective type of physical exercise.

This study aimed to review the effectiveness of different types of exercise intervention in executive function for older adults, to point out some gaps in existing research.

## Methods

### Search strategy

The current review was performed and reported according to Preferred Reporting Items for Systematic Reviews and Meta-Analyses (Page et al., 2021). We searched four electronic databases (PubMed, The Cochrane Library, Web of Science, and China National Knowledge Infrastructure) from inception to November 26, 2023. The search strategy employed a combination of subject terms and free-text terms. To avoid missing eligible literature, we reviewed the reference lists of systematic reviews published in the past 3 years and conducted a manual search to supplement relevant studies. The research strategy was as follows:

**Table:** 

#1 physical exercise OR physical activity OR aerobic exercise OR resistance exercise OR strength exercise OR stretching OR mind-body exercise OR flexibility exercise OR coordinative training OR multicomponent exercise #2 cognition OR executive function OR executive control #3 old people OR elderly OR old age OR the aged OR senior citizen OR older adults #4 #1 AND #2 AND #3

### Inclusion/exclusion criteria

Two researchers independently screened the records according to the predefined inclusion and exclusion criteria. Any discrepancies between the two authors were resolved by a third researcher upon reviewing the full texts.

The inclusion criteria were as follows: (1) participants had to be older adults; (2) interventions could encompass any type of exercise; (3) studies had to report at least one of the following outcomes: inhibition, working memory, switching, planning, and problem-solving; (4) the study design had to be randomized controlled trials (RCTs); (5) studies had to be written in either Chinese or English.

The exclusion criteria were as follows: (1) participants were older adults with dementia or mental disorders; (2) intervention programs included confounding factors, such as vitamin supplements and drugs; (3) data could not be extracted from the studies, even after contacting the authors; (4) studies were excluded if they were meta-analyses, reviews, case reports, degree papers, conference papers, or commentaries.

### Risk of bias assessment

Two researchers independently assessed the risk of bias in each study using the Cochrane Risk of Bias tool (Higgins et al., 2022). This tool comprised six domains assessing bias in RCTs: random sequence generation, allocation concealment, blinding of participants and personnel, blinding of outcome assessment, incomplete outcome data, and selective reporting. Any discrepancies were resolved through discussion with a third researcher until consensus was achieved.

### Data extraction

Two researchers independently extracted pertinent information from the studies, including author name, year of publication, participants’ characteristics (cognitive status, sample size, age, gender), details of the exercise prescription (type, duration, frequency, intensity, length), subdomains of executive function (inhibition, working memory, switching, planning), and main findings. In case of any discrepancies, a third researcher would be consulted, and consensus would be reached through discussion. If any data could not be extracted, researchers contacted the author(s) via email to request the missing information.

Exercise prescriptions were categorized into discrete variables. The type of exercise was classified as aerobic exercise (AE), strength exercise (STR), coordination exercise (COR), and mixed exercise (MIX) based on previous studies(Piercy et al., 2018; Ludyga et al., 2020). Frequency was classified into three levels: low (1–2 times per week), moderate (3–4 times per week), and high (5–7 times per week) (Zhang et al., 2023). Exercise intensity was categorized into five levels (refer to Table 1; Cai et al., 2021). Duration was classified as short (≤45 min), medium (46–60 min), or long (≥61 min) based on a previous systematic review (Northey et al., 2017). Length was divided into four categories: short-term (≤12 weeks), mid-term (13–24 weeks), long-term (25–48 weeks), and very long-term (≥49 weeks) (Zhang et al., 2023).

**Table 1:** Exercise intensity grading.

Intensity	%HRR	%HRmax	%VO2max	%1-RM	RPE
Very light	<30	<57	<37	<30	≤9
Light	≥30-<40	≥57-<64	≥37-<46	≥30-<50	9–10
Moderate	≥40-<60	≥64-<76	≥46-<64	≥50-<70	12–13
Vigorous	≥60-<90	≥76-<96	≥64-<91	≥70-<85	14–17
Near maximal to maximal	≥90	≥96	≥91	≥85	≥18

### Data synthesis

The descriptive information for all included studies was summarized in Supplementary Table S1. In cases where meta-analyses were not feasible or suitable, the effects of the studies were summarized based on the recommendation to synthesize and present the findings of the review (Mckenzie and Brennan, 2021). The proportions of positive and null/negative effects across all studies were calculated.

## Results

### Characteristics of included studies

**Figure 1:**
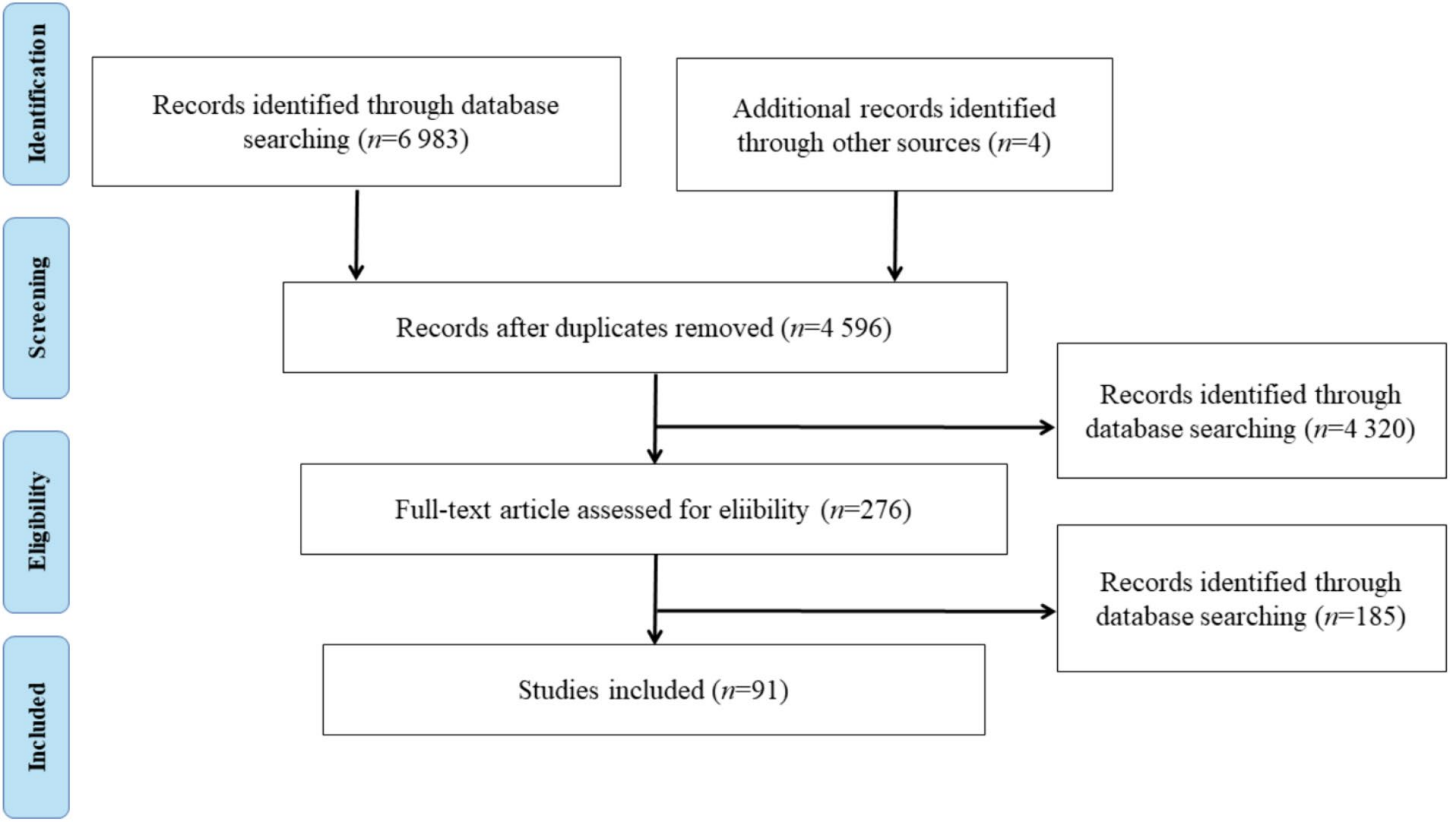
Literature selection flow diagram.

A total of 6,987 relevant articles were identified from the initial search. Following the removal of 2,387 duplicate articles, 4,596 articles remained after screening based on the title and abstract. A further review was conducted on the 276 potential full-text articles, resulting in the inclusion of 91 articles in the final review. Figure 1 depicted the flow of the literature search and study selection.

The characteristics of all 91 included studies were presented in Supplementary Table S1. The sample sizes ranged from 24 to 1,476, with an overall sample size of 9,482. Among the 91 included studies, 21 articles involved patients with MCI, while 71 articles involved cognitively healthy older adults (1 study included both MCI patients and healthy older adults). The average age ranged from 61 to 85.5 years across the studies. Most of the included studies (76.9%) had no sex-based restrictions, while 21 studies (23.1%) only included women as research subjects.

Supplementary Table S1 displayed four types of physical exercise identified in the 91 included studies (a study may contain two or more types of exercise): AE (*n* = 27), STR (*n* = 18), COR (*n* = 32), and MIX (*n* = 30). Among the 91 studies, 16 studies included two types of physical exercise. Exercise frequency ranged from one to seven times per week, with 3–4 times per week being the most common; exercise duration ranged from 30 min to 100 min, with 46–60 min being the most common; and exercise program length ranged from 4 weeks to 52 weeks, with 12–24 weeks being the most common. Exercise intensity measurement varied among studies, although most studies adopted moderate to vigorous intensity exercise. Among the 91 studies, 31 included a passive control group, while 60 included an active control group.

### Risk of bias analysis of included studies

**Figure 2:**
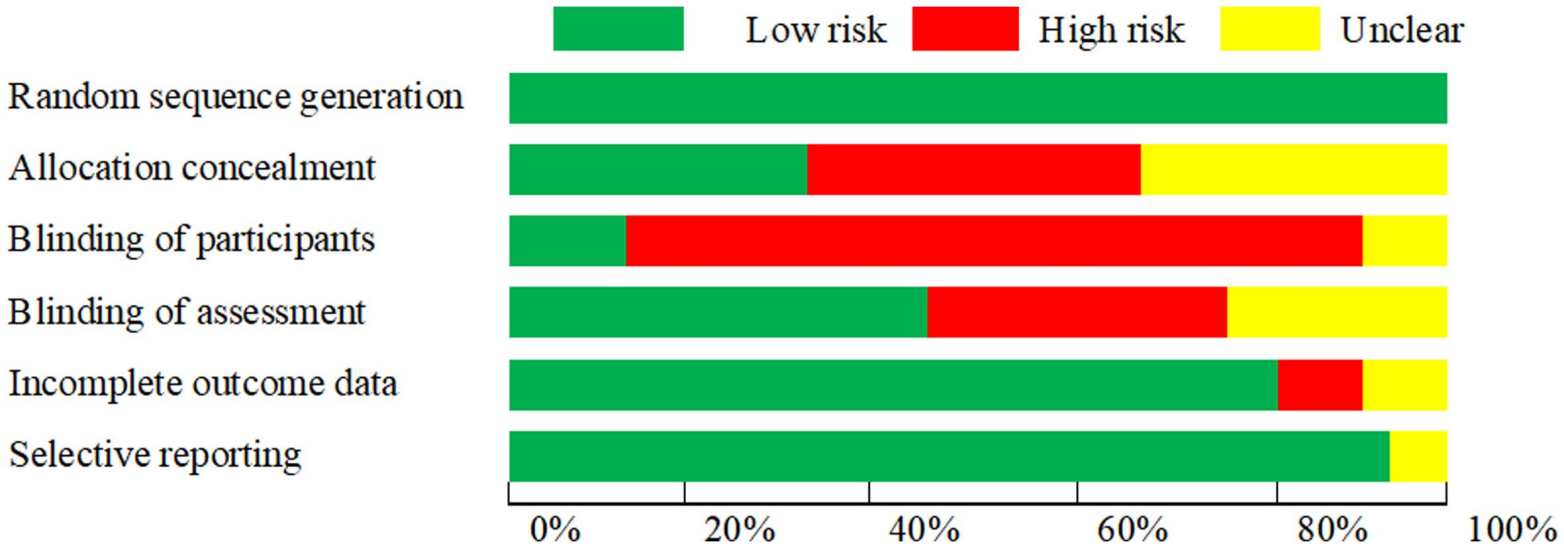
Risk of bias graph indicating the percentage of bias for all criteria in the included studies.

The risk of bias was categorized as high, medium, or low, expressed as a percentage for each item. Figure 2 presented the summary of the quality assessment data. Random sequence generation, incomplete outcome data, and selective reporting were assessed as low risk, while allocation concealment, blinding of participants, and blinding of assessment were assessed as high risk.

### Positive relationship between exercise prescription and executive function

Executive function is not a single structural system but rather a multidimensional cognitive structure (Miyake et al., 2000). The measurements for each subcomponent vary widely. The included studies utilized 10 task paradigms for inhibition, 18 task paradigms for working memory, 6 task paradigms for switching, and 7 task paradigms for planning. Owing to the significant heterogeneity in measuring executive function and exercise protocols, synthesizing the included data proved challenging. Consequently, we classified the exercise protocols and executive function measurements and conducted a descriptive analysis.

**Figure 3:**
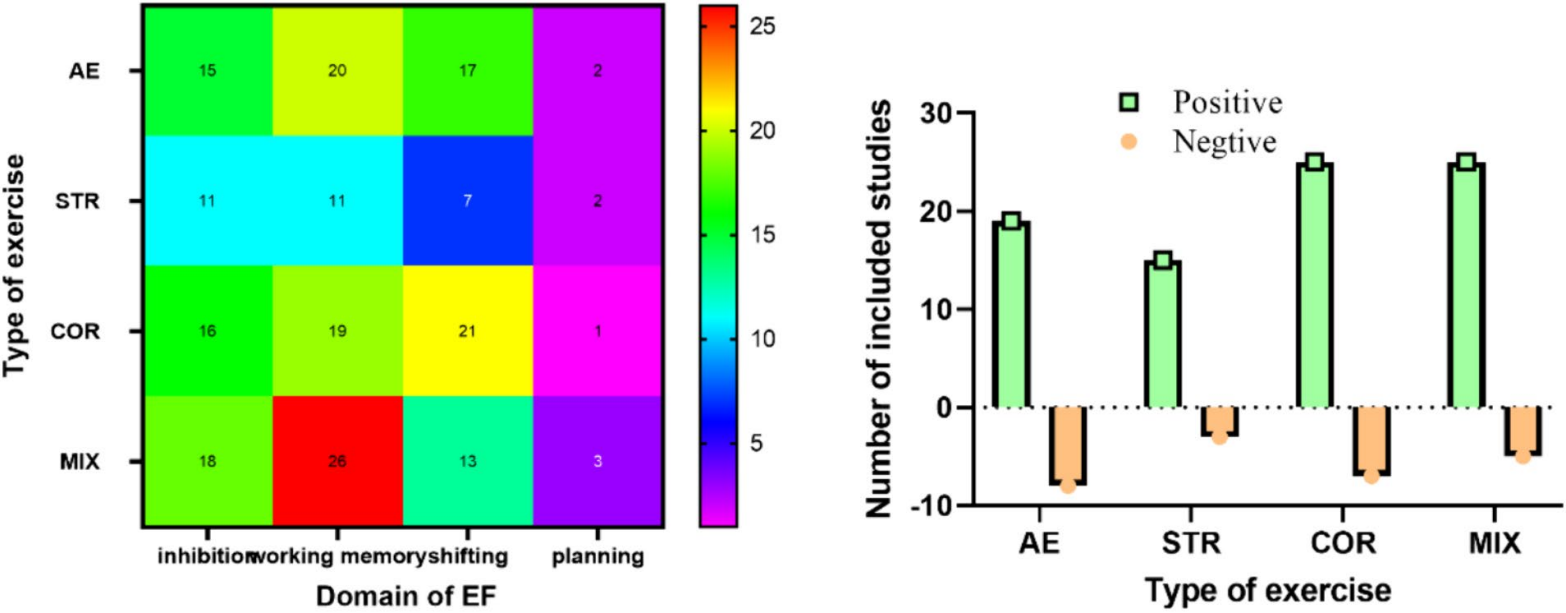
Illustration depicting types of exercise. One study can measure more than one domain of EF.

We summarized the results of the included studies in Figure 3. Among the 91 included studies, 27 (29.7%) utilized AE interventions targeting the executive function of older adults; among these, 19 (70.4%) reported positive effects, while 8 (29.7%) indicated negative or negligible effects. Eighteen studies (19.8%) implemented STR interventions targeting the executive function of older adults, with 15 studies (83.3%) reporting positive benefits and three studies (16.7%) indicating negative or negligible benefits. Thirty-two studies (35.2%) employed COR interventions targeting the executive function of older adults, with 25 studies (78.1%) reporting positive benefits and 7 studies (21.9%) indicating negative or negligible benefits. Thirty studies (33%) utilized MIX to intervene in the executive function of older adults, with 25 studies (82.3%) reporting positive benefits and 7 studies (16.7%) indicating negative or negligible benefits. Only a few included studies directly compared the intervention effects of different types of exercise.

The effects of each study on the sub-domain of executive function were presented in Figure 3. The highest proportion of positive effects were observed with MIX, followed by STR, COR, and lastly AE. Working memory was the focus of 83.5% of the included studies, followed by inhibition (65.9%), switching (63.7%), and planning (8.8%).

**Figure 4:**
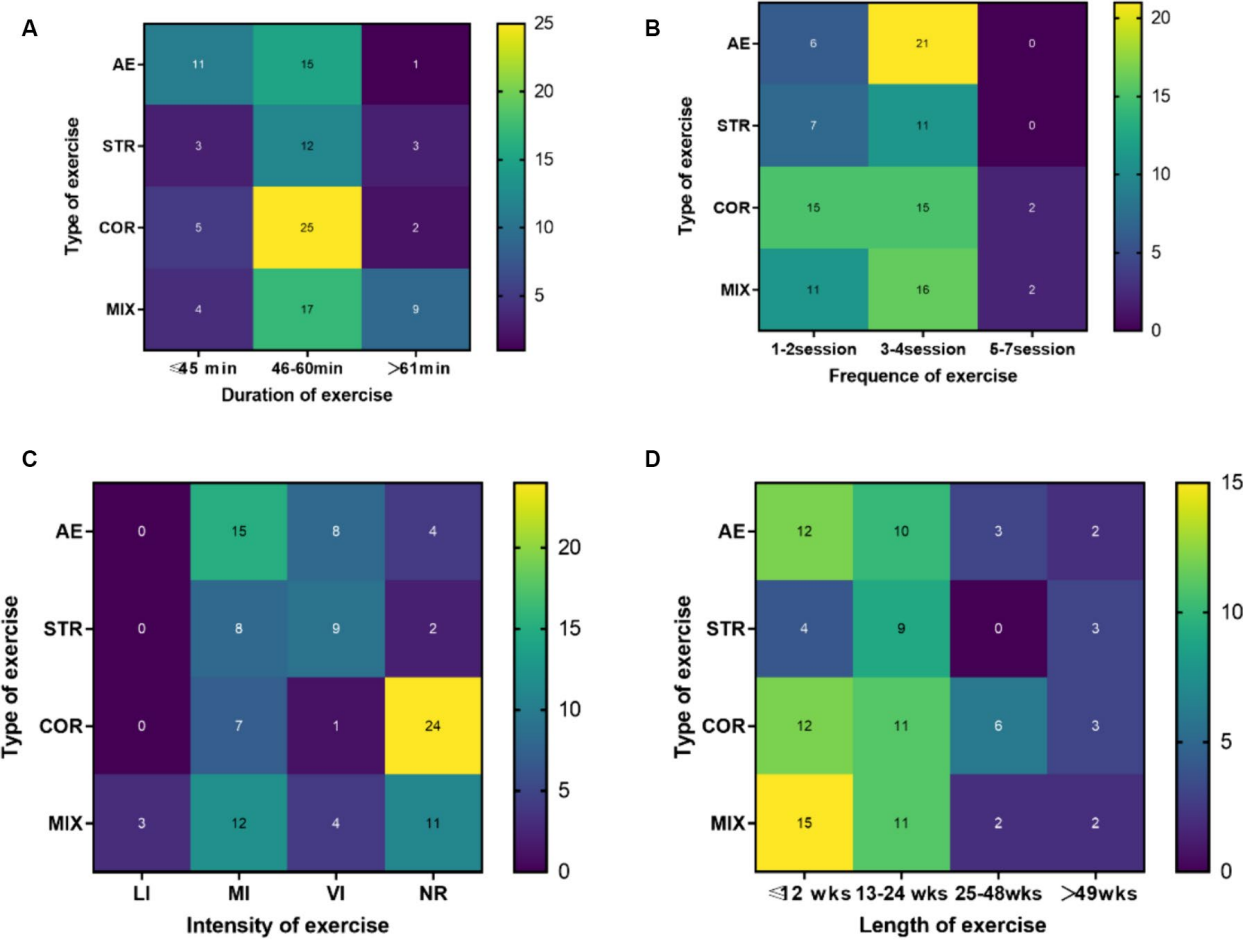
Illustration depicting exercise prescription Note: LI, light intensity; MI, moderate intensity; VI, vigorous intensity; NR, no report; AE, aerobic exercise; STR, strength exercise; COR, coordination exercise; MIX, mixed exercise; one study can measure more than one domain of executive function.

Out of the total 91 studies, 48 studies (52.8%) reported assigning older adults to a moderate frequency intervention (3–4 sessions weekly), 36 studies (39.6%) to a low frequency intervention (1–2 sessions weekly), and only 7 studies (7.7%) to a high frequency intervention (5–7 sessions weekly) (Figure 4).

Forty studies (44%) indicated that the exercise training intervention lasted a short-term length of time (≤12 weeks), 36 studies (40%) indicated a mid-term length (13–24 weeks), 8 studies (8.8%) indicated a long-term length (25–48 weeks), and 7 study (7.7%) indicated a very long-term length (≥49 weeks) (Figure 4).

The intensity measurements of the included studies varied, including heart rate reserve (HRR), maximum heart rate (HRmax), maximum oxygen uptake (VO2max), repetition maximum (RM), ventilatory anaerobic threshold of oxygen uptake, and anaerobic threshold. Moderate intensity was predominantly utilized in studies employing AE as an intervention, while high intensity was primarily employed in studies utilizing strength training. Conversely, the majority of studies employing coordinated movement as an intervention did not report exercise intensity (Figure 4).

Fifty-seven studies (62.6%) utilized moderate-duration (46–60 min) exercise sessions, with 60 min being the most frequently employed. Nineteen studies (20.9%) employed short-duration (≤45 min) exercise sessions, while 13 studies (14.3%) utilized longer session times (≥61 min) for exercise training. Three studies did not specify the timing of exercise training (Figure 4).

## Discussion

Our research and selection process yielded 91 studies, among which 27 studies investigated the effects of AE on executive function in older adults, 18 studies focused on STR, 32 studies examined COR, and 30 studies explored MIX. The current results confirmed that all four types of exercise had significant positive effects on executive function in older adults. MIX showed the highest likelihood of improving executive function in older adults, followed by STR, COR, and AE.

The results of this study are consistent with previous findings (Levin et al., 2017). Compared to a non-exercise control group, a combination of aerobic and strength training was more effective in mitigating cognitive decline in older adults (Bossers et al., 2015). Combourieu et al. demonstrated significant enhancements in executive function across the three training programs, with cognitive and physical training proving superior to the other two groups (Combourieu Donnezan et al., 2018). In contrast to single-component exercises, MIX comprises various elements, including aerobic exercise, strength training, stretching, among others. These components may complement each other, enhancing cognitive benefits by eliciting favorable biochemical changes through various neurobiological mechanisms. Furthermore, in addition to the complementary effects of multiple motor components, research has shown that MIX can directly enhance executive function through cognitive stimulation inherent in the varied motor tasks involving perceptual-motor adaptation and diverse neuromuscular coordination (Forte et al., 2013). Thus, MIX appears to be the most effective approach.

Our study yielded an intriguing finding: STR exhibited a higher likelihood of enhancing executive function in older adults compared to COR and AE. While the effectiveness of AE in enhancing executive function has been validated by numerous studies (Zheng et al., 2016; Castells-Sánchez et al., 2022), recent research suggests that STR may be more effective than AE (Cherup et al., 2018; Li et al., 2018; Huang et al., 2022). In a previous network meta-analysis, resistance exercise was identified as the most efficient modality, followed by exergames, aerobic exercise, and mind–body exercise (Wang et al., 2019). Although both AE and STR are repetitive and highly metabolic, they exert distinct physiological effects. AE primarily enhances cardiovascular health, whereas STR predominantly increases muscle mass and strength. Therefore, AE and STR may contribute to brain function through divergent and shared biological pathways.

The superior efficacy of STR over AE in enhancing executive function in older adults may be attributed to its multifaceted enhancement of cognitive function. Initially, STR enhances cognition by elevating insulin-like growth factor 1 (IGF-1) levels in both the hippocampus and peripheral blood (Tsai et al., 2019). Secondly, enhancing muscle strength and function via STR can additionally stimulate the secretion of irisin, subsequently elevating IGF-1 and brain-derived neurotrophic factor (BDNF) levels, mitigating oxidative stress, fostering neurogenesis, and enhancing insulin sensitivity (Kim et al., 2015). Thirdly, STR may exert beneficial effects on the brain and cognition through the modulation of inflammatory cytokines released during muscle contraction, including interleukin 6 (IL-6), IL-1b, and IL-15 (Nielsen and Pedersen, 2007).

AE was initially the exercise type investigated for its impact on cognitive function (Netz, 2019). Numerous studies have reported that aerobic exercise elevates BDNF levels. Erickson et al. discovered that following 1 year of aerobic exercise intervention, the experimental group exhibited an 11% increase in BDNF levels and a 2% increase in hippocampal volume (Erickson et al., 2011). Leckie et al. observed that engaging in 40 min of daily walking for 12 months notably enhanced both BDNF levels and executive function. Conversely, the control group experienced a decrease in BDNF levels (Leckie et al., 2014).

Recently, an expanding body of research has elucidated the advantages of COR on executive function in older adults, encompassing practices such as Tai Chi, yoga, Baduanjin, exergames, and dance. COR shares similarities with MIX in its ability to enhance cardiovascular fitness, muscle strength, coordination, and social interaction, all of which contribute to the enhancement of cognitive function.

A review demonstrated that the high cognitive demands inherent in COR directly impact cognitive function (Netz, 2019). COR emphasizes coordinated movement of the body and rhythmic breathing, emphasizing the integration of mind and body, which demands more cognitive resources. For instance, participants must memorize movement skills and consistently recall movement patterns and sequences, processes that contribute to the enhancement of working memory and attention allocation. Research indicated that both STR and COR can enhance executive function. However, mediation analysis revealed distinct mechanisms: STR indirectly enhances executive function by enhancing muscle strength, whereas COR directly influences executive function (Forte et al., 2013). In another study comparing the impacts of Tai Chi and walking on executive function, both exercise groups exhibited enhanced Stroop reaction times, yet the Tai Chi group demonstrated superior accuracy rates (Ji et al., 2017).

Since there are few articles directly comparing the effects of different types of exercise in the included studies, caution is warranted when interpreting the aforementioned findings. Per the guidelines from the American College of Sports Medicine, exercise prescriptions encompass exercise frequency, intensity, duration, and modality (Piercy et al., 2018). Crucially, the type of exercise may interact with other prescription factors, including duration, intensity, frequency, and length, to modulate the extent of benefit on executive function. Previous systematic reviews have identified moderators that influence the benefits on executive function (Zhidong et al., 2021).

## Limitations

Firstly, exercise protocols exhibit wide variation, and the classification of exercise types may be subject to flaws. Secondly, articles examining the effects of physical activity interventions combined with other interventions (e.g., vitamin supplements) were excluded. This exclusion might have led to the omission of significant evidence concerning multicomponent or real-world interventions. Thirdly, we refrained from conducting a meta-analysis. Instead, we employed a form of “vote counting” to document the proportion of between-group comparisons yielding positive results.

## Conclusion

While all types of exercise can enhance executive function in older adults, encompassing inhibition, working memory, shifting, and planning, their effectiveness may vary. Combining different types of exercise may yield a synergistic effect. We have identified a research gap in directly comparing the effects of various types of exercise on executive function. Future experimental studies should aim to directly compare the effects of different types of exercise interventions.

## Data availability statement

The original contributions presented in the study are included in the article/Supplementary material, further inquiries can be directed to the corresponding author/s.

## Author contributions

ZC: Writing – original draft, Writing – review & editing. RC: Writing – review & editing. SL: Writing – original draft, Writing – review & editing.
