# Supplementary material for: Effects of different types of physical exercise on executive function of older adults: a scoping review
Source: Front Psychol. 2024 Jun 28;15:1376688. doi: 10.3389/fpsyg.2024.1376688 (PMC11239569; doi:10.3389/fpsyg.2024.1376688)
Supplement: Supplementary file 1 [file Table_1.docx]

Table 1. Overview of included studies that examined effects of physical exercise on executive function in older adults

|  | Cognitive health | Sample | Gender | Age | Type | Grouping | T | F | L | Intensity | EF domain and task | Main finding |
| --- | --- | --- | --- | --- | --- | --- | --- | --- | --- | --- | --- | --- |
| (Albinet et al., 2010) | Healthy | 24 | both | 70.7 | END | I: walking, running; C: stretching | 60 | 3 | 12 | MI（40%-60%HRR） | WCST: inhibition | the participants in the aerobic training group improved their performance on the WCST. |
|  | Healthy | 36 | both | 67 | END | I: swimming; C: stretching | 60 | 2 | 21 | VI(40%-60%) | Stroop, RNG, The Hayling task: inhibition; 2-back, Verbal/Spatial Running Span: working memory; The Dimension-switching task, Digit -letter task, The plus-minus task: switching | the participants in the aquaerobics group significantly improved their performance for the Stroop test and the verbal running-span test at the end of the program. |
| (Albinet et al., 2016) | Healthy | 69 | both | 82.4 | MIX(END, STR, BFE), STR | I: cycling, strengthening; C: no intervention | 60 | 3 | 16 | VI(60%~85% HRR) | CDT: planning; Verbal fluency: working memory | There were no significant differences between groups and times in any of the variables. |
| (Antunes et al., 2015) | Healthy | 51 | both | 66.9 | END, MIX | I: dancing and recreational and handcraft activities, running; C: no intervention | 60 | 3 | 24 | VI | WCST:inhibition; DS, ROCF,Letter fluency: working memory | (END)physical exercise improves neuropsychological function. |
| (Bae et al., 2019) | MCI | 83 | both | 76 | MIX | I: walking, strengthening, stretching, TaiChi, dancing; C: social activity | 90 | 2 | 24 | NR | TMT: shifting; Corsi block-tapping task: working memory | the intervention group significantly improve the spatial working memory compared with the control group. |
| (Baker et al., 2010) | MCI | 29 | both | 67 | END | I: treadmill; C: stretching | 55 | 4 | 24 | VI(75%-85%HRR) | TMT, Task switching: shifting; Verbal Fluency: working memory | high-intensity aerobic exercise had sex-specific effects on EF. |
| (Baniqued et al., 2017) | Healthy | 96 | both | 65.2 | END， COR | I: walking, dancing; C: stretching | 60 | 3 | 24 | MI(50%-75%HRmax) | task-switching: shifting; spatial working memory: working memory | END and COR improved EF. |
| (Berryman et al., 2014) | Healthy | 52 | both | 70.6 | MIX(END, STR), BFE | I: strengthening, cycling; C: stretching | 60 | 2 | 8 | MI-VI(60%-100% maximal aerobic power) | Stroop, RNG: inhibition; DS: working memory | All groups showed equivalent improvement in inhibition. |
| (Best et al., 2015) | Healthy | 155 | both | 69.7 | STR | I: strengthening; C: balance-and-toning | 60 | 3 | 16 | MI(80%~100%1RM) | Stroop: inhibition; TMT: shifting; DS: working memory | Both frequencies of resistance training promoted executive function compared to balance-and-toning |
| (Blumenthal et al., 1991) | Healthy | 101 | both | 67 | END, COR | I: cycling, yoga; C: no intervention | 60 | 3 | 16 | MI(70%HRR) | TMT: shifting; Stroop: inhibition; DS, Verbal Fluency Test: working memory | there were relatively few improvements in cognitive performance associated with aerobic exercise. |
| (Boa Sorte Silva et al., 2018) | MCI | 127 | both | 67.5 | MIX(END, STR, BFE) | I: strengthening; balance, cycling; C: balance | 60 | 3 | 24 | MI-VI(65%~85% HR­max) | CBS: planning, working memory | There were no significant differences between groups in EF, but significant differences were observed after follow-up favoring between groups. |
| (Bossers et al., 2015) | De | 109 | both | 85.5 | MIX, END | I： strengthening, walking; C: social visit | 30 | 4 | 9 | MI-VI(50%-85%HRmax) | Stroop: inhibition; DS, verbal fluency: working memory, TMT: shifting | Compared to a non-exercise control group, a combination of aerobic and strength training is more effective than aerobic-only training in slowing cognitive decline in patients with dementia. |
| (Bouaziz et al., 2019) | Healthy | 60 | both | 73.6 | END | I: cycling; C: no intervention | 30 | 2 | 10 | VI | TMT: shifting; Verbal Fluency Test: working memory | Compared to controls, aerobic training improved TMT. |
| (Brown et al., 2009) | Healthy | 154 | both | 79.6 | MIX | I: walking, strengthening, balance, flexibility; C: no intervention | 60 | 2 | 24 | NR | TMT: shifting; DS: working memory; COWAT, Stroop: inhibition | MIX significantly improved cognitive performance of fluid intelligence compared with the control group, but did not improve EF. |
| (Cassilhas et al., 2007) | Healthy | 62 | male | 68 | STR | I: strengthening; C: placebo activity | 60 | 3 | 24 | MI(50%1RM),HI(80%1RM) | DS, Rey-Osterrieth Complex Figure: working memory | Moderate- and high-intensity resistance exercise programs had equally beneficial effects on EF. |
| (S. B. Chapman et al., 2013) | Healthy | 37 | both | 64 | END | I: bike and treadmill; C: no intervention | 60 | 3 | 12 | MI(50%-75%HRmax) | TMT: shifting; DS: working memory; DKEFS-color word: inhibition | The exercise group improved immediate and delayed memory performance from baseline to post-training but did not improve EF. |
| (Sandra B. Chapman et al., 2016) | Healthy | 55 | both | 63.5 | END, CT | I: cycling; C: cognitive training | 60 | 3 | 12 | MI(50%-75%HRmax) | COWAT,Daneman Carpenter,Verbal fluency: working memory; DKEFS,TMT:shifting | The CT group improved on executive function whereas the END group’s memory was enhanced. |
| (Cherup et al., 2018) | Healthy | 30 | female | 69.3 | END, STR | I: treadmill, strengthening; C: no intervention | 35 | 3 | 12 | END: MI(55%HRR), STR: VI | NIH Cognitive Toolbox: inhibition, working memory, shifting | The results support the use of STR over END for improving EF in older persons |
| (Coetsee & Terblanche, 2017) | Healthy | 46 | both | 62.7 | STR | I: strengthening, walking; C: no intervention | 60 | 3 | 16 | VI(75%,85%,100% 10RM) | Stroop: inhibition | STR improved executive cognitive function |
| (Combourieu Donnezan et al., 2018) | MCI | 32 | both | 76.8 | END,MIX | I: cycling, cognition training; C: no intervention | 60 | 2 | 12 | LI-MI(60%HRmax) | DS: working memory; Stroop: inhibition; Matrix Reasoning test: planning | the results revealed significant improvements in executive control in the three training programs, the PCT (cognitive and physical training ) is better than the other two groups. |
| (Damirchi et al., 2018) | MCI | 54 | female | 68.4 | MIX: END, STR | I: walking, cognition training; C: no intervention | 60 | 3 | 8 | MI-VI(END,55%~75% HRR, STR, 13~15RPE) | DS: working memory; Stroop: inhibition | The positive effect of mental training on the cognitive parameters is parallel with BDNF elevation. |
| (Dao et al., 2013) | Healthy | 73 | female | 69.4 | STR | I: strengthening; C: stretching | 60 | 1 or 2 | 52 | VI(80%-100%1RM) | Stroop: inhibition | the experiment group improved the Stroop performance. |
| (Davis et al., 2013) | MCI | 56 | female | 74.9 | STR, END | I: strengthening, walking; C: stretching, balance | 60 | 2 | 24 | NR | Stroop: inhibition | STR had significantly improved executive function. |
| (Desjardins-Crépeau et al., 2016) | Healthy | 76 | both | 72.3 | MIX(END, STR, COE) | I: strengthening, walking; C: cognition training | 60 | 2 | 12 | MI | TMT: shifting; Color-Word Interference Test: inhibition | Combined training significantly improved executive function. |
| (Doi et al., 2017) | MCI | 134 | both | 76 | COR | I: dancing; C: health education | 60 | 1 | 40 | NR | TMT:shifting | dance group showed improvement in memory and general cognitive function compared with the health education group, but unimprovement in EF. |
| (Eggenberger et al., 2015) | Healthy | 71 | both | 78.9 | MIX: END, STR, BFE | I: dancing, walking, strengthening; C: cognition | 60 | 3 | 24 | 5-7RPE | TMT: shifting; EC, DS: working memory | Particular executive functions benefit from simultaneous cognitive–physical training compared to exclusively physical multicomponent training. |
| (Eggenberger et al., 2016) | Healthy | 33 | both | 74.9 | COR | I: dancing; C: strengthening | 30 | 3 | 8 | NR | EC: working memory; TMT: shifting; Stroop: inhibition | These exercise training-induced modulations in PFC oxygenation correlated with improved executive functions. |
| (Eggermont et al., 2009) | MCI | 97 | both | 85.4 | END | I: walking; C: social activity | 30 | 3 | 6 | NR | DS, letter fluency: working memory; category fluency: shifting | No positive effects on cognition were found. |
| (Emery & Gatz, 1990) | Healthy | 48 | both | 72 | MIX | I: walking, strengthening, stretching; C: social activity | 60 | 3 | 12 | MI(70%HRmax) | DS: working memory | there was very little change in any of the cognitive measures in either group. |
| (Kirk I. Erickson et al., 2011) | Healthy | 120 | both | 66.6 | END | I: walking; C: stretching | 40 | 3 | 52 | MI(50%-75%HRmax) | Spatial Memory Task: working memory | aerobic exercise training is effective at reversing hippocampal volume loss in late adulthood, which is accompanied by improved memory function. |
| (Esmail et al., 2020) | Healthy | 41 | both | 67.5 | END, MIX | I: dancing, cycling; C: no intervention | 60 | 3 | 12 | MI | N-back: working memory; Stroop: inhibition | Dance/Movement Training may have a positive impact on EF. |
| (Eyre et al., 2017) | MCI | 79 | both | 68 | COR | I: Yoga; C: cognition training | 60 | 1 | 12 | NR | TMT: shifting; Visuospatial ability: working memory; Stroop: inhibition | the yoga group showed short- and long-term improvements in executive functioning as compared to the Memory training group. |
| (Fabre et al., 2002) | Healthy | 32 | both | 65.9 | END | I: cycling, cognition training; C: leisure activities | 60 | 1 | 8 | MI | DS: working memory | combined training seemed to lead to greater effects than either technique alone. |
| (Ferreira et al., 2015) | Healthy | 68 | both | 67.5 | END | I: walking, respiratory training; C: social interaction | 45 | 3 | 24 | MI( 60%–80%HRR) | WCST: inhibition | the interventions used yielded positive results for the cognitive functions of the elderly. |
| (Fiatarone Singh et al., 2014) | Healthy | 100 | both | ＞50 | STR, MIX | I: strengthening; C: sham physical training | 75 | 2~3 | 24 | 8reps | Matrices, WAIS, COWAT(verbal fluency): working memory | The progressive STR improved EF. |
| (Fogarty et al., 2016) | MCI | 40 | both | 72 | COR | I:TaiChi; C: cognition | 90 | 2 | 10 | NR | TMT: shifting; DS: working memory | No preferential benefit was found for individuals in the MIP + TTC group on cognition. |
| (Gajewski & Falkenstein, 2018) | Healthy | 141 | both | 70.9 | MIX: END, STR | I: cycling, strengthening; C: no intervention | 90 | 2 | 16 | NR | Task-switching: shifting; N-back: working memory | A greater improvement in performance for attendants of the cognitive training group compared to the other groups. |
| (Gothe et al., 2014) | Healthy | 118 | both | 62 | COR | I: Yoga; C: stretching, strengthening | 60 | 3 | 8 | NR | running memory span, N-back: working memory; Task-switching: shifting | participants in the yoga intervention group showed significantly improved performance on the executive function measures of working memory capacity and efficiency of mental set shifting and flexibility compared with their stretching–strengthening counterparts. |
| (Gothe et al., 2016) | Healthy | 118 | both | 62 | COR | I: Yoga; C: stretching, strengthening | 60 | 3 | 8 | NR | running memory span, N-back: working memory; Task-switching: shifting | Yoga participants showed improved accuracy in executive function |
| (Hariprasad et al., 2013) | Healthy | 87 | both | 75.3 | COR | I: Yoga; C: no intervention | 60 | 1 | 24 | NR | DS, Special span, COWA: working memory; Stroop: inhibition; TMT: shifting | The yoga-based‑intervention appears beneficial to improve several domains of cognitive function in the elderly. |
| (Hong et al., 2018) | MCI, Healthy | 56 | both | 75.3 | STR | I: strengthening; C: no intervention | 60 | 2 | 12 | MI(65%1RM) | DS: working memory; Stroop: inhibition | The DS test for working memory was significantly changed in the MCI group. |
| (Iuliano et al., 2015) | Healthy | 80 | both | 67 | END, STR, COR | I: strengthening, cycling, postural and balance exercise; C: no intervention | 40 | 3 | 12 | MI(80-85%1RM,70-80%HRR) | Stroop: inhibition; TMT: shifting; RPM: planning | STR significantly improved the EF of older adults. |
| (L. Ji et al., 2017) | Healthy | 141 | both | 70.9 | MIX: END, STR, BFE, MBE | I: walking, strengthening, yoga, balance; C: no intervention | 30 | 6 | 6 | NR | Stroop: inhibition; TMT: shifting; DS: working memory | Participants demonstrated a significant improvement in memory and executive function. |
| (Jonasson et al., 2017) | Healthy | 58 | both | 68.8 | END | I: walking; C: stretching | 45 | 3 | 24 | MI( 40%–80%HRmax) | TMT, Odd Even Task: shifting; DS, N-back: working memory; Flanker Task: inhibition | aerobic exercisers, compared to controls, exhibited a broad, rather than specific, improvement in cognition. |
| (Kalbe et al., 2018) | Healthy | 35 | both | 68 | MIX | I: strengthening, cognition, coordination; C: cognitive training | 90 | 2 | 7 | NR | DS: working memory | all used training types are helpful to maintain cognition. |
| (Kimura et al., 2010) | Healthy | 119 | both | 74.4 | STR | I: strengthening; C: health education | 90 | 2 | 12 | MI(60%1RM) | Task Switching: shiting | this training period may not be sufficient to affect executive function in relatively healthy older people. |
| (Klusmann et al., 2010) | Healthy | 259 | female | 73.6 | MIX: END, STR, BFE | I: cycling, strengthening, flexibility; C: no intervention | 90 | 3 | 24 | NR | Stroop: inhibition; TMT: shifting; Verbal Fluency: working memory | The exercise group showed improved WM. |
| (Kosmat & Vranic, 2017) | Healthy | 24 | both | 79.8 | COR | I: dancing; C: placebo exercise | 45 | 1 | 10 | NR | WCST: inhibitIon | Training procedures, based on dance, could improve cognitive functioning in old-old |
| (Kramer, 2001) | Healthy | 124 | both | 66.6 | END | I: walking; C: stretching | 10-40 | 3 | 24 | VI(65%-70%VO2max) | task-switching: shifting; N-back, DS: working memory | The intervention group improved many sub-domain of EF. |
| (Lachman et al., 2006) | Healthy | 210 | both | 75.3 | STR | I: strengthening; C: no intervention | 30 | 3 | 24 | MI(10RM) | DS: working memory | strength training can benefit working memory among older adults, especially when using higher resistance levels. |
| (Lam et al., 2011) | MCI | 389 | both | 77.6 | COR | I: Tai Chi; C: stretching | 48 | 1 | 52 | NR | Category Fluency test, DS: working memory; TMT: shifting | the intervention group had greater improvement in many domains of EF. |
| (Lautenschlager et al., 2008) | Healthy | 29 | both | 81 | MIX | I: walking, strengthening; C: behavioral intervention | 60 | 3 | 12 | VI(75%-85%HRmax) | Verbal Frequency: working memory | physical activity provided a modest improvement in cognition. |
| (Lazarou et al., 2017) | MCI | 154 | both | 66.9 | COR | I: dancing; C: no intervention | 60 | 2 | 40 | NR | Verbal Frequency, ROCFT: working memory | Dance may be an important non-pharmacological approach that can benefit EF. |
| (Regina L. Leckie et al., 2014) | Healthy | 92 | both | 66.8 | END | I: walking; C: stretching | 40 | 3 | 48 | VI(60-74%HRR) | Task Switching: shifting | BDNF mediated the effect of the intervention on task-switch accuracy |
| (Legault et al., 2011) | Healthy | 36 | both | 76.4 | END | I: walking, cognition training C: cognition training | 50 | 3 | 14 | NR | Task-switching, TMT: shifting; N-back, self-ordered pointing task: working memory; Flanker task: inhibition | There were no statistically significant differences in 4-month changes in EF. |
| (Liu et al., 2019) | Healthy | 62 | both | 61 | COR | I: Tai Chi, Baduanjin; C: health education | 60 | 5 | 12 | NR | DS: working memory | both intervention groups improved the cognition of older adults. |
| (Liu-Ambrose et al., 2010) | Healthy | 155 | both | 69.6 | STR | I: strengthening; C: balance-and-toning | 60 | 1 or 2 | 52 | HI | Stroop: inhibition; TMT: shifting; DS: working memory | Progressive resistance training once- or biweekly improved selective attention and conflict resolution. |
| (Liu-Ambrose et al., 2012) | Healthy | 52 | both | 69.3 | STR | I: strengthening; C: balance-and-toning | 60 | 1 or 2 | 52 | VI | Flanker: inhibition | resistance training improved flanker task performance. |
| (X. Lu et al., 2016) | Healthy | 31 | female | 70.1 | COR | I: Yang style Tai Chi; C: general interest classes | 90 | 3 | 16 | NR | Stroop: inhibition | Tai Chi subjects made fewer errors in the auditory Stroop test under dual-task condition after Tai Chi training. |
| (Lü et al., 2016) | MCI | 79 | female | 69.7 | STR, COR | I: strengthening; C: no intervention | 60 | 3 | 16 | NR | Stroop: inhibition; TMT: shifting; DS: working memory | There was a significant within-group improvement in TMTand BDS scores. |
| (Maki et al., 2012) | Healthy | 150 | both | 72 | END | I: walking; C: health education | 90 | 1 | 12 | NR | TMT: shifting; Visuospatial abilities, verbal fluency: working memory | Significant differences between the intervention and control groups were shown in word fluency. |
| (Marmeleira et al., 2009) | Healthy | 32 | both | 68.3 | MIX | I: walking; C: no intervention | 60 | 3 | 12 | NR | TMT: shifting; Stroop: inhibition | No significant improvement was shown in EF. |
| (Mavros et al., 2017) | MCI | 100 | both | ＞55 | STR | I: strengthening; C: stretching | 60~100 | 2~3 | 24 | VI(80%~92%1RM) | Category fluency: working memory | STR Group has a trend for improvement in executive function. |
| (Merom, Grunseit, et al., 2016) | Healthy | 115 | both | 69.5 | COR | I: dancing; C: walking | 60 | 2 | 32 | NR | TMT:shifting; WCST:inhibition; DS: working memory | The superior potential of dance over walking on executive functions of cognitively healthy and active older adults was not supported |
| (Merom, Mathieu, et al., 2016) | Healthy | 530 | both | 78 | COR | I: dancing; C: no intervention | 60 | 2 | 40 | NR | TMT: shifting | Dance intervention didn't significantly improve the TMT. |
| (Mortimer et al., 2012) | Healthy | 120 | both | 67.8 | COR | I: Tai Chi, walking; C: no intervention | 50 | 3 | 40 | NR | Verbal fluency, DS: working memory; TMT: shifting; Stroop: inhibition | Compared to the no Intervention group, Tai Chi group significantly improved in TMT, Verbal Fluency, and increased brain volume. |
| (Nagamatsu et al., 2012) | Healthy | 86 | female | 67.8 | STR, END | I: strengthening, walking; C: balance and tone | 60 | 2 | 24 | VI(6-8RM, 70%~80%HRR) | Stroop: inhibition; TMT: shifting | STR group significantly improved performance on the Stroop Test. |
| (Nguyen & Kruse, 2012) | Healthy | 96 | both | 68.8 | COR | I: Tai Chi; C: no intervention | 60 | 2 | 24 | NR | TMT: shifting | Participants in the Tai chi group reported significant improvement in TMT in comparison with the control group. |
| (Nishiguchi et al., 2015) | Healthy | 48 | both | 73.3 | MIX | I: walking, strengthening; C: no intervention | 90 | NR | 12 | MI | TMT: shifting | Exercise group participants had significantly greater postintervention improvement in memory and executive functions than the control group. |
| (Norouzi et al., 2019) | Healthy | 64 | both | 68.3 | MIX | I: strengthening, motor training, cognition training; C: informal meetings | 60-80 | 3 | 4 | NR | Corsi block-tapping task: working memory | Dual-task interventions improved working memory, but more so if cognitive performance was specifically trained along with resistance training |
| (Nouchi et al., 2014) | Healthy | 61 | both | 66.9 | MIX | I: strengthening, running, stretching; C: no intervention | 30 | 3 | 4 | MI(60-80HRmax) | Stroop: inhibition; DS, Verbal Fluency Test: working memory | the combination of exercise training improved executive functions, episodic memory, and processing speed compared to the control group. |
| (Oken et al., 2006) | Healthy | 135 | both | 72.1 | END, COR | I: yoga, walking; C: no intervention | 60 | 1 | 24 | MI(RPE6-7) | Stroop: inhibition | There were no relative improvements in cognitive function among healthy seniors in the yoga or exercise group compared to the wait-list control group. |
| (Riegle van West et al., 2018) | Healthy | 96 | both | 68.4 | COR | I: Yang style Tai Chi; C: Poi | 60 | 2 | 4 | NR | Stroop: inhibition; SA: shifting | Poi seems to be as effective as Tai Chi for improving physical and cognitive function in healthy older adults. |
| (Scherder et al., 2005) | MCI | 30 | both | 85 | END | I: walking; C: social activity | 30 | 3 | 6 | NR | DS: working memory; TMT, Category Naming: shifting | a (nearly) significant improvement in tasks appealing to EF was observed in both the walking group and the hand/face group compared to the control group. |
| (Shatil, 2013) | Healthy | 122 | both | 76.8 | MIX | I: aerobic training, cognition training; C: reading | 45 | 3 | 16 | NR | WCST, Stroop: inhibition; Global Visual Memory: working memory | The combined training group showed significant improvement in Global Visual Memory, Visual Scanning, and Naming. |
| (Sink et al., 2015) | Healthy | 1476 | both | 67.7 | MIX: END, STR, BFE | I: walking, strength, flexibility, and balance training; C: health education | 50 | 5 | 8 | NR | task-switching, TMT: shifting; N-back, ROCF: working memory; Stroop: inhibition | Participants in the physical activity group who were 80 years or older had better changes in executive function composite scores compared with the health education group. |
| (Smiley-Oyen et al., 2008) | Healthy | 105 | both | 70.2 | END, MIX | I: aerobic exercise equipment; C: strength, flexibility, and balance exercises | 50 | 3 | 40 | VI(65%-85%HRR) | Stroop, Go/No-Go Task, WCST: inhibition | Aerobic exercise in older adults can have a beneficial effect on the performance of speeded tasks that rely heavily on executive control. |
| (Sun et al., 2015) | Healthy | 138 | both | 69.2 | COR | I: Yang Style Tai Chi; C: playing cards or singing | 60 | 2 | 6 | NR | FAB: inhibition | Tai Chi practice may improve cognitive in the elderly. |
| (Sungkarat et al., 2018) | MCI | 66 | both | 67.8 | COR | I: Tai Chi; C: health education | 50 | 3 | 24 | NR | TMT: shifting; DS: working memory | TC training significantly improved memory and the mental switching component of executive function in older adults with a-MCI, possibly via upregulation of BDNF. |
| (Suzuki et al., 2013) | MCI | 50 | both | 75 | MIX | I: strengthening, circuit training; C: health education | 90 | 2 | 48 | LI(60%HRmax) | VFT, Category fluency: working memory; Stroop: inhibition | multicomponent exercise improved cognitive performance in older adults with MCI. |
| (Tao et al., 2017) | Healthy | 63 | both | 61.6 | COR | I: Yang style Tai Chi, Baduanjin; C: no intervention | 60 | 5 | 12 | NR | DS: working memory | We found that compared to healthy controls, Tai Chi Chuan and Baduanjin significantly improved EF and significantly increased grey matter volume. |
| (Taylor-Piliae et al., 2010) | Healthy | 132 | both | 69.1 | MIX(END, STR, BFE), COR | I: Tai Chi, walking, strengthening, flexibility; C: attention control | 60 | 1-2d | 52 | MI | verbal fluency, DS: working memory | Tai Chi had greater improvements in EF than MIX and the control group. |
| (Tsai et al., 2017) | Healthy | 64 | both | 66.2 | END, COR | I: walking, cycling, tennis; C: stretching | 30 or 40 | 3 | 24 | VI(70-75% HRmax) | Task Switching: shifting; N-back: working memory | the two exercise modes produced different levels of neuropsychologically beneficial effects on the task-switching and the N-back task. |
| (Vaughan et al., 2014) | Healthy | 49 | female | 68.9 | MIX: END, STR, BFE | I: cardiovascular training, strengthening, balance; C: no intervention | 60 | 2 | 16 | LI(RPE3-6) | TMT: shifting; Stroop: inhibition; LNS: working memory | The intervention group performed significantly better than the control group in the Trail Making test A/B and Stroop |
| (Voelcker-Rehage et al., 2011) | Healthy | 44 | both | 69.6 | END, COR | I: walking, coordination training; C: stretching | 60 | 3 | 52 | MI | Flanker Task: inhibition | In both intervention groups, prefrontal areas showed decreased activation after 6 and 12 months when performing an executive control task, as compared to the control group. |
| (Walsh et al., 2015) | Healthy | 60 | both | 64.2 | COR | I: Yang, Wu, Chen, or Sun style Tai Chi C: usual care | 30 | 2 | 24 | NR | DS: working memory; TMT: shifting | TC training did not significantly improve any measures of cognitive function. |
| (Williamson et al., 2009) | Healthy | 102 | both | 77.4 | MIX | I: walking, strengthening, balance, flexibility exercise; C: health education | 75 | 2 | 52 | NR | Stroop: inhibition | physical exercise has a positive effect on EF. |
| (Wu et al., 2018) | Healthy | 31 | both | 64.9 | COR | I: Yang style Tai Chi; C: no intervention | 60 | 3 | 12 | NR | Task swichting: shifting | the TCC group showed decreased errors in task-switching performance and increased prefrontal activation increases in the switch condition. |
| (Xia et al., 2019) | MCI | 69 | both | 65.5 | COR | I: Tai Chi; C1: walking, C2: no intervention | 60 | 3 | 24 | NR | Stroop: inhibition | Baduanjin exercise significantly increased the EF (inhibition) of MCI patients. |
| (Yang et al., 2019) | Healthy | 26 | female | 65 | COR | I: Tai Chi, walking; C: no intervention | 45 | 3 | 8 | NR | Flanker Task: inhibition | Tai Chi group improved the flanker task performance and increased oxy-Hb in the PFC. |
| (Yoon et al., 2018) | MCI | 43 | both | 73.9 | STR | I: stretching; C: stretching | 60 | 3 | 16 | MI(RPR 12 to 13) | DS: working memory | Exercise significantly improved EF performance. |
| (Yin et al., 2022) | Healthy/MCI | 25/25 | both | 72.06 | MIX | I:jogging; strengthening; balance;flexibility | 60 | 3 | 20 | VI(55%-75% HRmax) | DS: working memory; TMT: shifting | Multi-mode exercise improved the TMT performance. |

Note: F= frequency; T=time; L= length; LI= very light intensity; MI=moderate intensity; VI= vigorous intensity; HI=high intensity; CDT=Clock Drawing task; RST= running span task; WAIS-Ⅲ=Wechsler Adult Intelligence Scale 3rd edition; DKEFS=Delis-Kaplan Executive Function System Sorting Test; COWAT= Controlled Oral Word Association Test; CT=Cognitive training; LNS= letter number sequencing; RPM=Raven's Progressive Matrices Tests; IR= Image recall; SA= Shifting attention; FAB=Frontal assessment battery; WCST=Wisconsin Card Sort Test; ROCFT= Rey-Osterrieth Complex Figure Test; COWA=Controlled Oral Word Association; CBS= Cambridge Brain Sciences cognitive battery; RNG= Random number generation; TMT= Trail making test; DS= Digit span; SA= Shifting attention; EC= Executive control

**1.** Albinet CT, Boucard G, Bouquet CA, Audiffren M. Increased heart rate variability and executive performance after aerobic training in the elderly. *Eur. J. Appl. Physiol.* 2010;109(4):617-624.

**2.** Albinet CT, Abou-Dest A, Andre N, Audiffren M. Executive functions improvement following a 5-month aquaerobics program in older adults: Role of cardiac vagal control in inhibition performance. *Biol. Psychol.* 2016;115:69-77.

**3.** Ansai JH, Rebelatto JR. Effect of two physical exercise protocols on cognition and depressive symptoms in oldest-old people: A randomized controlled trial. *Geriatrics & Gerontology International.* 2015;15(9):1127-1134.

**4.** Antunes HK, Santos-Galduroz RF, De Aquino Lemos V, et al. The influence of physical exercise and leisure activity on neuropsychological functioning in older adults. *Age (Dordrecht, Netherlands).* 2015;37(4):9815.

**5.** Bae S, Lee S, Lee S, et al. The effect of a multicomponent intervention to promote community activity on cognitive function in older adults with mild cognitive impairment: A randomized controlled trial. *Complement. Ther. Med.* 2019;42:164-169.

**6.** Baker LD, Frank LL, Foster-Schubert K, et al. Effects of Aerobic Exercise on Mild Cognitive Impairment A Controlled Trial. *Arch. Neurol.* 2010;67(1):71-79.

**7.** Baniqued PL, Gallen CL, Voss MW, et al. Brain Network Modularity Predicts Exercise-Related Executive Function Gains in Older Adults. *Front. Aging Neurosci.* 2017;9:426.

**8.** Berryman N, Bherer L, Nadeau S, et al. Multiple roads lead to Rome: combined high-intensity aerobic and strength training vs. gross motor activities leads to equivalent improvement in executive functions in a cohort of healthy older adults. *Age (Dordrecht, Netherlands).* 2014;36(5):9710.

**9.** Best JR, Chiu BK, Hsu CL, Nagamatsu LS, Liu-Ambrose T. Long-Term Effects of Resistance Exercise Training on Cognition and Brain Volume in Older Women: Results from a Randomized Controlled Trial. *J. Int. Neuropsychol. Soc.* 2015;21(10):745-756.

**10.** Blumenthal JA, Emery CF, Madden DJ, et al. Long-term effects of exercise on psychological functioning in older men and women. *J. Gerontol.* 1991;46(6):352-361.

**11.** Boa Sorte Silva NC, Gill DP, Owen AM, et al. Cognitive changes following multiple-modality exercise and mind-motor training in older adults with subjective cognitive complaints: The M4 study. *PLoS One.* 2018;13(4):e0196356.

**12.** Bossers WJR, van der Woude LHV, Boersma F, Hortobagyi T, Scherder EJA, van Heuvelen MJG. A 9-Week Aerobic and Strength Training Program Improves Cognitive and Motor Function in Patients with Dementia: A Randomized, Controlled Trial. *Am. J. Geriatr. Psychiatry.* 2015;23(11):1106-1116.

**13.** Bouaziz W, Schmitt E, Vogel T, et al. Effects of a short-term Interval Aerobic Training Programme with active Recovery bouts (IATP-R) on cognitive and mental health, functional performance and quality of life: A randomised controlled trial in sedentary seniors. *Int. J. Clin. Pract.* 2019;73(1):e13219.

**14.** Brown AK, Liu-Ambrose T, Tate R, Lord SR. The effect of group-based exercise on cognitive performance and mood in seniors residing in intermediate care and self-care retirement facilities: a randomised controlled trial. *Br. J. Sports Med.* 2009;43(8):608-614.

**15.** Cassilhas RC, Viana VA, Grassmann V, et al. The impact of resistance exercise on the cognitive function of the elderly. *Med. Sci. Sports Exerc.* 2007;39(8):1401‐1407.

**16.** Chapman SB, Aslan S, Spence JS, et al. Shorter term aerobic exercise improves brain, cognition, and cardiovascular fitness in aging. *Front. Aging Neurosci.* 2013;5:75.

**17.** Chapman SB, Aslan S, Spence JS, et al. Distinct Brain and Behavioral Benefits from Cognitive vs. Physical Training: A Randomized Trial in Aging Adults. *Front. Hum. Neurosci.* 2016;10(338).

**18.** Cherup N, Roberson K, Potiaumpai M, et al. Improvements in cognition and associations with measures of aerobic fitness and muscular power following structured exercise. *Exp. Gerontol.* 2018;112:76-87.

**19.** Coetsee C, Terblanche E. The effect of three different exercise training modalities on cognitive and physical function in a healthy older population. *European Review of Aging and Physical Activity.* 2017;14(1):13.

**20.** Combourieu Donnezan L, Perrot A, Belleville S, Bloch F, Kemoun G. Effects of simultaneous aerobic and cognitive training on executive functions, cardiovascular fitness and functional abilities in older adults with mild cognitive impairment. *Mental Health and Physical Activity.* 2018;15:78-87.

**21.** Damirchi A, Hosseini F, Babaei P. Mental Training Enhances Cognitive Function and BDNF More Than Either Physical or Combined Training in Elderly Women With MCI: A Small-Scale Study. *American Journal of Alzheimers Disease and Other Dementias.* 2018;33(1):20-29.

**22.** Dao E, Davis JC, Sharma D, Chan A, Nagamatsu LS, Liu-Ambrose T. Change in Body Fat Mass Is Independently Associated with Executive Functions in Older Women: A Secondary Analysis of a 12-Month Randomized Controlled Trial. *PLoS One.* 2013;8(1):1-6.

**23.** Davis JC, Bryan S, Marra CA, et al. An economic evaluation of resistance training and aerobic training versus balance and toning exercises in older adults with mild cognitive impairment. *PLoS One.* 2013;8(5):e63031.

**24.** Desjardins-Crépeau L, Berryman N, Fraser SA, et al. Effects of combined physical and cognitive training on fitness and neuropsychological outcomes in healthy older adults. *Clin. Interv. Aging.* 2016;11:1287-1299.

**25.** Doi T, Verghese J, Makizako H, et al. Effects of Cognitive Leisure Activity on Cognition in Mild Cognitive Impairment: Results of a Randomized Controlled Trial. *J. Am. Med. Dir. Assoc.* 2017;18(8):686-691.

**26.** Eggenberger P, Schumacher V, Angst M, Theill N, de Bruin ED. Does multicomponent physical exercise with simultaneous cognitive training boost cognitive performance in older adults? A 6-month randomized controlled trial with a 1-year follow-up. *Clin. Interv. Aging.* 2015;10:1335-1349.

**27.** Eggenberger P, Wolf M, Schumann M, de Bruin ED. Exergame and Balance Training Modulate Prefrontal Brain Activity during Walking and Enhance Executive Function in Older Adults. *Front. Aging Neurosci.* 2016;8(66):1-16.

**28.** Eggermont LHP, Swaab DF, Hol EM, Scherder EJA. Walking the line: a randomised trial on the effects of a short term walking programme on cognition in dementia. *Journal of Neurology Neurosurgery & Psychiatry.* 2009;80(7):802-804.

**29.** Emery CF, Gatz M. Psychological and cognitive effects of an exercise program for community-residing older adults. *The Gerontologist.* 1990;30(2):184-188.

**30.** Erickson KI, Voss MW, Prakash RS, et al. Exercise training increases size of hippocampus and improves memory. *Proc. Natl. Acad. Sci. U. S. A.* 2011;108(7):3017-3022.

**31.** Esmail A, Vrinceanu T, Lussier M, et al. Effects of Dance/Movement Training vs. Aerobic Exercise Training on cognition, physical fitness and quality of life in older adults: A randomized controlled trial. *J. Bodyw. Mov. Ther.* 2020;24(1):212-220.

**32.** Eyre HA, Siddarth P, Acevedo B, et al. A randomized controlled trial of Kundalini yoga in mild cognitive impairment. *Int. Psychogeriatr.* 2017;29(4):557-567.

**33.** Fabre C, Chamari K, Mucci P, Masse-Biron J, C P. Improvement of Cognitive Function by Mental and/or Individualized Aerobic Training in Healthy Elderly Subjects. *Int. J. Sports Med.* 2002;23(06):415-421.

**34.** Ferreira L, Tanaka K, Santos-Galduroz RF, Fernandes Galduroz JC. Respiratory training as strategy to prevent cognitive decline in aging: a randomized controlled trial. *Clin. Interv. Aging.* 2015;10:593-603.

**35.** Fiatarone Singh MA, Gates N, Saigal N, et al. The Study of Mental and Resistance Training (SMART) study—resistance training and/or cognitive training in mild cognitive impairment: a randomized, double-blind, double-sham controlled trial. *J. Am. Med. Dir. Assoc.* 2014;15(12):873-880.

**36.** Fogarty JN, Murphy KJ, McFarlane B, et al. Taoist Tai Chi® and Memory Intervention for Individuals with Mild Cognitive Impairment. *Journal of aging and physical activity.* 2016;24(2):169‐180.

**37.** Gajewski PD, Falkenstein M. ERP and Behavioral Effects of Physical and Cognitive Training on Working Memory in Aging: A Randomized Controlled Study. *Neural Plast.* 2018;2018:3454835.

**38.** Gothe NP, Kramer AF, McAuley E. The Effects of an 8-Week Hatha Yoga Intervention on Executive Function in Older Adults. *Journals of Gerontology Series a-Biological Sciences and Medical Sciences.* 2014;69(9):1109-1116.

**39.** Gothe NP, Keswani RK, McAuley E. Yoga practice improves executive function by attenuating stress levels. *Biol. Psychol.* 2016;121:109-116.

**40.** Hariprasad VR, Koparde V, Sivakumar PT, et al. Randomized clinical trial of yoga-based intervention in residents from elderly homes: Effects on cognitive function. *Indian J. Psychiatry.* 2013;55(Suppl 3):S357-S363.

**41.** Hong S-G, Kim J-H, Jun T-W. Effects of 12-Week Resistance Exercise on Electroencephalogram Patterns and Cognitive Function in the Elderly With Mild Cognitive Impairment: A Randomized Controlled Trial. *Clin. J. Sport Med.* 2018;28(6):500-508.

**42.** Iuliano E, di Cagno A, Aquino G, et al. Effects of different types of physical activity on the cognitive functions and attention in older people: A randomized controlled study. *Exp. Gerontol.* 2015;70:105-110.

**43.** Ji L, Zhang H, Potter GG, et al. Multiple Neuroimaging Measures for Examining Exercise-induced Neuroplasticity in Older Adults: A Quasi-experimental Study. *Front. Aging Neurosci.* 2017;9:102.

**44.** Jonasson LS, Nyberg L, Kramer AF, Lundquist A, Riklund K, Boraxbekk C-J. Aerobic Exercise Intervention, Cognitive Performance, and Brain Structure: Results from the Physical Influences on Brainin Aging (PHIBRA) Study. *Front. Aging Neurosci.* 2017;8(336):1-15.

**45.** Kalbe E, Roheger M, Paluszak K, et al. Effects of a Cognitive Training With and Without Additional Physical Activity in Healthy Older Adults: A Follow-Up 1 Year After a Randomized Controlled Trial. *Front. Aging Neurosci.* 2018;10.

**46.** Kimura K, Obuchi S, Arai T, et al. The Influence of Short-term Strength Training on Health-related Quality of Life and Executive Cognitive Function. *J. Physiol. Anthropol.* 2010;29(3):95-101.

**47.** Klusmann V, Evers A, Schwarzer R, et al. Complex mental and physical activity in older women and cognitive performance: a 6-month randomized controlled trial. *J. Gerontol. A Biol. Sci. Med. Sci.* 2010;65(6):680-688.

**48.** Kosmat H, Vranic A. The Efficacy of a Dance Intervention as Cognitive Training for the Old-Old. *J Aging Phys Act.* 2017;25(1):32-40.

**49.** Kramer AF, Hahn, S., McAuley, E. , Cohen, N. J.,. *Exercise，aging andcognition: Healthy body，healthy mind．*. Mahwah2001.

**50.** Lachman ME, Neupert SD, Bertrand R, Jette AM. The effects of strength training on memory in older adults. *Journal of Aging and Physical Activity.* 2006;14(1):59-73.

**51.** Lam LC, Chau RC, Wong BM, et al. Interim follow-up of a randomized controlled trial comparing Chinese style mind body (Tai Chi) and stretching exercises on cognitive function in subjects at risk of progressive cognitive decline. *Int. J. Geriatr. Psychiatry.* 2011;26(7):733‐740.

**52.** Lautenschlager NT, Cox KL, Flicker L, Foster JK, Almeida OP. Effect of Physical Activity on Cognitive Function in Older Adults at Risk for Alzheimer Disease: A Randomized Trial. *J. Am. Med. Assoc.* 2008;300(9):1027-1037.

**53.** Lazarou I, Parastatidis T, Tsolaki A, et al. International Ballroom Dancing Against Neurodegeneration: A Randomized Controlled Trial in Greek Community-Dwelling Elders With Mild Cognitive impairment. *Am. J. Alzheimers Dis. Other Demen.* 2017;32(8):489-499.

**54.** Leckie RL, Oberlin LE, Voss MW, et al. BDNF mediates improvements in executive function following a 1-year exercise intervention. *Front. Hum. Neurosci.* 2014;8:985.

**55.** Legault C, Jennings JM, Katula JA, et al. Designing clinical trials for assessing the effects of cognitive training and physical activity interventions on cognitive outcomes: the Seniors Health and Activity Research Program Pilot (SHARP-P) study, a randomized controlled trial. *BMC Geriatr.* 2011;11:27.

**56.** Liu J, Tao J, Liu W, et al. Different modulation effects of Tai Chi Chuan and Baduanjin on resting-state functional connectivity of the default mode network in older adults. *Soc. Cogn. Affect. Neurosci.* 2019;14(2):217-224.

**57.** Liu-Ambrose T, Nagamatsu LS, Graf P, Beattie BL, Ashe MC, Handy TC. Resistance Training and Executive Functions A 12-Month Randomized Controlled Trial. *Arch. Intern. Med.* 2010;170(2):170-178.

**58.** Liu-Ambrose T, Nagamatsu LS, Voss MW, Khan KM, Handy TC. Resistance training and functional plasticity of the aging brain: a 12-month randomized controlled trial. *Neurobiol. Aging.* 2012;33(8):1690-1698.

**59.** Lu X, Siu KC, Fu SN, Hui-Chan CW, Tsang WW. Effects of Tai Chi training on postural control and cognitive performance while dual tasking - a randomized clinical trial. *Journal of complementary & integrative medicine.* 2016;13(2):181-187.

**60.** Lü J, sun M, liang l, Feng Y, Pan X, liu Yu. Effects of momentum-based dumbbell training on cognitive function in older adults with mild cognitive impairment: a pilot randomized controlled trial. *Clin. Interv. Aging.* 2016;11(1):9-16.

**61.** Maki Y, Ura C, Yamaguchi T, et al. Effects of intervention using a community-based walking program for prevention of mental decline: a randomized controlled trial. *J. Am. Geriatr. Soc.* 2012;60(3):505-510.

**62.** Marmeleira JF, Godinho MB, Fernandes OM. The effects of an exercise program on several abilities associated with driving performance in older adults. *Accid. Anal. Prev.* 2009;41(1):90-97.

**63.** Mavros Y, Gates N, Wilson GC, et al. Mediation of Cognitive Function Improvements by Strength Gains After Resistance Training in Older Adults with Mild Cognitive Impairment: Outcomes of the Study of Mental and Resistance Training. *J. Am. Geriatr. Soc.* 2017;65(3):550-559.

**64.** Merom D, Grunseit A, Eramudugolla R, Jefferis B, McNeill J, Anstey KJ. Cognitive Benefits of Social Dancing and Walking in Old Age: The Dancing Mind Randomized Controlled Trial. *Frontiers in aging neuroscience.* 2016;8:26.

**65.** Merom D, Mathieu E, Cerin E, Morton RL, Simpson JM, Rissel C. Social Dancing and Incidence of Falls in Older Adults: A Cluster Randomised Controlled Trial. *PLoS Med.* 2016;13(8):e1002112.

**66.** Mortimer JA, Ding D, Borenstein AR, et al. Changes in brain volume and cognition in a randomized trial of exercise and social interaction in a community-based sample of non-demented Chinese elders. *Journal of Alzheimer's disease : JAD.* 2012;30(4):757-766.

**67.** Nagamatsu L, Handy T, Hsu CL, et al. Resistance training promotes cognitive functions and functional plasticity in senior women with probable mild cognitive impairment: a 6-month randomized controlled trial. *Alzheimer's & dementia.* 2012;8(4):522-523.

**68.** Nguyen MH, Kruse A. A randomized controlled trial of Tai chi for balance, sleep quality and cognitive performance in elderly Vietnamese. *Clin. Interv. Aging.* 2012;7:185-190.

**69.** Nishiguchi S, Yamada M, Tanigawa T, et al. A 12-Week Physical and Cognitive Exercise Program Can Improve Cognitive Function and Neural Efficiency in Community-Dwelling Older Adults: A Randomized Controlled Trial. *J. Am. Geriatr. Soc.* 2015;63(7):1355-1363.

**70.** Norouzi E, Vaezmosavi M, Gerber M, Puhse U, Brand S. Dual-task training on cognition and resistance training improved both balance and working memory in older people. *Physician Sportsmed.* 2019.

**71.** Nouchi R, Taki Y, Takeuchi H, et al. Four weeks of combination exercise training improved executive functions, episodic memory, and processing speed in healthy elderly people: evidence from a randomized controlled trial. *Age.* 2014;36(2):787-799.

**72.** Oken BS, Zajdel D, Kishiyama S, et al. Randomized, controlled, six-month trial of yoga in healthy seniors: effects on cognition and quality of life. *Altern. Ther. Health Med.* 2006;12(1):40-47.

**73.** Riegle van West K, Stinear C, Buck R. The Effects of Poi on Physical and Cognitive Function in Healthy Older Adults. *Journal of aging and physical activity.* 2018:1-9.

**74.** Scherder EJA, Paasschen JV, Deijen JB, Knokke SVD, Sergeant JA. Physical activity and executive functions in the eldery with mild cognitive impairment. *Aging & Mental Health.* 2005;9(3):272-280.

**75.** Shatil E. Does combined cognitive training and physical activity training enhance cognitive abilities more than either alone? A four-condition randomized controlled trial among healthy older adults. *Front. Aging Neurosci.* 2013;5.

**76.** Sink KM, Espeland MA, Castro CM, et al. Effect of a 24-Month Physical Activity Intervention vs Health Education on Cognitive Outcomes in Sedentary Older Adults: The LIFE Randomized Trial. *J. Am. Med. Assoc.* 2015;314(8):781-790.

**77.** Smiley-Oyen AL, Lowry KA, Francois SJ, Kohut ML, Ekkekakis P. Exercise, Fitness, and Neurocognitive Function in Older Adults: The “Selective Improvement” and “Cardiovascular Fitness” Hypotheses. *Ann. Behav. Med.* 2008;36(3):280-291.

**78.** Sun J, Kanagawa K, Sasaki J, Ooki S, Xu H, Wang L. Tai chi improves cognitive and physical function in the elderly: a randomized controlled trial. *Journal of physical therapy science.* 2015;27(5):1467-1471.

**79.** Sungkarat S, Boripuntakul S, Kumfu S, Lord SR, Chattipakorn N. Tai Chi Improves Cognition and Plasma BDNF in Older Adults With Mild Cognitive Impairment: A Randomized Controlled Trial. *Neurorehab. Neural Repair.* 2018;32(2):142-149.

**80.** Suzuki T, Shimada H, Makizako H, et al. A Randomized Controlled Trial of Multicomponent Exercise in Older Adults with Mild Cognitive Impairment. *PLoS One.* 2013;8(4):1-10.

**81.** Tao J, Chen X, Egorova N, et al. Tai Chi Chuan and Baduanjin practice modulates functional connectivity of the cognitive control network in older adults. *Sci. Rep.* 2017;7.

**82.** Taylor-Piliae RE, Newell KA, Cherin R, Lee MJ, King AC, Haskell WL. Effects of Tai Chi and Western exercise on physical and cognitive functioning in healthy community-dwelling older adults. *Journal of aging and physical activity.* 2010;18(3):261-279.

**83.** Tsai C-L, Pan C-Y, Chen F-C, Tseng Y-T. Open- and Closed-Skill Exercise Interventions Produce Different Neurocognitive Effects on Executive Functions in the Elderly: A 6-Month Randomized, Controlled Trial. *Front. Aging Neurosci.* 2017;9(294):1-16.

**84.** Vaughan S, Wallis M, Polit D, Steele M, Shum D, Morris N. The effects of multimodal exercise on cognitive and physical functioning and brain-derived neurotrophic factor in older women: a randomised controlled trial. *Age Ageing.* 2014;43(5):623-629.

**85.** Voelcker-Rehage C, Godde B, Staudinger U. Cardiovascular and Coordination Training Differentially Improve Cognitive Performance and Neural Processing in Older Adults. *Front. Hum. Neurosci.* 2011;5.

**86.** Walsh JN, Manor B, Hausdorff J, et al. Impact of Short- and Long-term Tai Chi Mind-Body Exercise Training on Cognitive Function in Healthy Adults: Results From a Hybrid Observational Study and Randomized Trial. *Global advances in health and medicine.* 2015;4(4):38-48.

**87.** Williamson JD, Espeland M, Kritchevsky SB, et al. Changes in cognitive function in a randomized trial of physical activity: results of the lifestyle interventions and independence for elders pilot study. *Journal of Gerontology: MEDICAL SCIENCES.* 2009;10(10):688-694.

**88.** Wu MT, Tang PF, Goh JOS, et al. Task-Switching Performance Improvements After Tai Chi Chuan Training Are Associated With Greater Prefrontal Activation in Older Adults. *Frontiers in Aging Neuroscience.* 2018;10:280.

**89.** Xia R, Qiu P, Lin H, et al. The Effect of Traditional Chinese Mind-Body Exercise (Baduanjin) and Brisk Walking on the Dorsal Attention Network in Older Adults With Mild Cognitive Impairment. *Front. Psychol.* 2019;10:2075.

**90.** Yang Y, Chen T, Shao M, Yan S, Yue GH, Jiang C. Effects of Tai Chi Chuan on Inhibitory Control in Elderly Women: An fNIRS Study. *Front. Hum. Neurosci.* 2019;13:476.

**91.** Yoon DH, Lee JY, Song W. Effects of Resistance Exercise Training on Cognitive Function and Physical Performance in Cognitive Frailty : a Randomized Controlled Trail *Journal of Nutrition Health & Aging.* 2018;22(8):944-951.
